# Fasting and Cognitive Impairment

**DOI:** 10.3390/nu15245108

**Published:** 2023-12-14

**Authors:** Luca Tagliafico, Alessio Nencioni, Fiammetta Monacelli

**Affiliations:** 1 Section of Geriatrics, Department of Internal Medicine and Medical Specialties (DIMI), University of Genoa, 16132 Genoa, Italy; alessio.nencioni@unige.it (A.N.); fiammetta.monacelli@unige.it (F.M.); 2 IRCCS Ospedale Policlinico San Martino, 16132 Genoa, Italy

Fasting is a nutritional practice involving complete food restriction for a varying length of time. Mounting evidence shows numerous possible beneficial effects of this type of dietary regimen in terms of health, but also shows the possible risks, including malnutrition [1]. Several approaches to fasting have been established in the attempt to obtain its potential benefits in terms of disease prevention or treatment, while minimizing the risks associated with it. Given the need to alternate periods of fasting (or severe dietary restriction) with periods of regular eating, these approaches are typically referred to as intermittent fasting (IF) [1]. Daily IF, which is one of the most commonly adopted solutions, restricts daily eating to one six- to eight-hour period each day (this type of IF is also referred to as “time-restricted feeding”). For instance, the classical 16/8 fasting scheme foresees eating for eight hours and fasting for sixteen. Alternatively, one can undergo alternate day fasting or use the 5:2 IF approach, which involves eating normally for five days a week, while limiting food intake to one 500–600 calorie meal/day during the other two days. Fasting for longer periods (e.g., for three days or longer) but less frequently (e.g., once a month) is a form of IF that is sometimes referred to as “periodic fasting”. The prolonged abstinence from food that the latter type of IF foresees is harder to tolerate compared with other IF approaches. Thus, several “modified fasting” regimens have been reported that can be used to implement periodic fasting (instead of undergoing “water-only” fasting). These regimens typically consist of very low-calorie, low-protein, low-sugar vegan diets that last a few days and induce the metabolic effects of water-only fasting. Typically, a modified fasting regimen provides between 300 and 1000 kcal day, lasts for a few days (e.g., four-to-seven days) and is repeated every few weeks (e.g., monthly). Among these modified fasting regimens there are also medical food kits, such as the so called “fasting-mimicking diet” (FMD) by L-Nutra Inc. (a spin-off of the University of Southern California), which are specifically developed to achieve the putative benefits of IF while maximizing the tolerance of these diets [2]. Overall, the different forms of IF have shown an ability to improve the health span and also to prevent several aging-related diseases, including AD, in animal models [2]. Thus, there is now a considerable interest in assessing whether IF will also be useful for the prevention and treatment of such conditions.

The beneficial effects of IF have been attributed to several mechanisms, including the reduction of reactive oxygen species (ROS), the elimination of senescent cells and the removal of misfolded proteins through autophagy [3]. Studies also suggest that IF may have a significant impact on age-related mitochondrial dysfunction [4], which could contribute to the reduction in tissue ROS [5]. This reduction in oxidative stress appears to reflect increased antioxidant enzyme activity and an enhanced turnover of oxidized proteins [5]. Regarding the increased turnover of oxidized or misfolded proteins, extensive evidence supports the idea that fasting enhances autophagy, likely through AMPK activation and mTOR inhibition [6].

Another vital aspect associated with the beneficial effects of IF is its impact on the immune system. Fasting was shown to reduce markers of inflammation and to counteract the chronic low-grade inflammation (often referred to as “inflammaging”) that is strongly correlated with age-associated diseases [7,8]. Fasting was also shown to reduce markers of cellular senescence and thus to possibly help to slow cellular senescence [9]. This partial elimination of senescent cells through fasting is also associated with evidence of tissue regeneration, particularly during the refeeding phase following fasting, often with the involvement of tissue stem cells [10].

All these beneficial effects of fasting, which have been reported in mice and still await confirmation in humans, are also of great interest in research related to neurodegenerative diseases, including AD [11]. In this area, most data still come from preclinical studies, which have yielded promising results both in terms of biological markers of disease progression and of clinical outcomes [12,13,14].

Brandhorst and colleagues showed that IF cycles (with L-Nutra’s FMD) trigger neurogenesis, as shown by an increased proliferation of immature neurons and an increase in the expression of NeuroD1 (a transcription factor important for neuron protection and differentiation) [12]. These effects were observed in radial glia-like cells (type I) and non-radial precursor (type II) neural stem cells, in immature neurons, and in the dendrite-covered area. Strikingly, these changes were associated with a clear improvement in motor coordination, hippocampal-dependent learning, and short- and long-term memory [12]. Interestingly, a previous study by this group showed that protective and potentially rejuvenating effects can also be achieved in mice with a non-calorie-restricted but protein-restricted periodic diet (seven days of this low-protein diet followed by 7 days of normal, ad libitum eating) with the supplementation of non-essential amino acids [13]. In mice already displaying significant cognitive impairment and AD-like pathology, this protein-restricted diet decreased tau phosphorylation in the hippocampus and alleviated age-dependent cognitive impairment. More recently, these findings were further confirmed in a study by Priya Rangan and colleagues, where the IF (again, using the FMD) led to improved clinical outcomes in two mouse models of AD (E4FAD and 3xTg) [14]. Specifically, the study demonstrated a reduction in hippocampal Aβ load and hyperphosphorylated tau, accompanied by improvements in cognitive tests. Veerendra Kumar Madala Halagappa and colleagues demonstrated that IF improved cognitive function in a mouse model of AD (3xTgAD), albeit without a significant reduction in hippocampal amyloid beta (Aβ)1-40 and Aβ1-42 levels [15]. This led to the hypothesis that the clinical benefits of IF might be primarily related to neuronal protection [15]. Another study by Jingzhu Zhang and colleagues showed cognitive improvement through IF in association with reduced amyloid plaques in the APP/PS1 mouse model of AD [16]. This improvement was linked to the restoration of AQP4 polarity, resulting in enhanced Aβ clearance [16]. Similarly, the study by Bae Kun Shin and colleagues demonstrated that IF favorably affected amyloid load and cognitive function, in addition to enhancing the metabolic status, in a rat AD model [17]. However, it is worth noting that in this model IF caused a loss of bone mineral density and increased insulin resistance, which are both common pathological conditions in older adults with AD [17]. Other studies have shown that a possible mediator of the beneficial effects of IF against AD may be the microbiome and the gut–brain axis [18]. A recent study by Daniel S. Whittaker and colleagues shows several beneficial effects of time-restricted feeding in another AD model (APP23 TG), such as the modulation of hippocampal gene expression with respect to AD-related pathways and inflammation, an increase in Aβ clearance and a reduction in amyloid deposition and an improvement in circadian rhythm and cognitive function [19].

On the other hand, in other studies, such as the one conducted by Divna Lazic and colleagues, IF failed to produce beneficial effects in terms of clinical disease progression and pathophysiological processes [20]. The discrepancies observed in different studies can be partly attributed to differences in the IF regimens and in the mouse AD models that were utilized. Nevertheless, these differences underscore the need to conduct additional preclinical studies to define the real benefit of fasting-based dietary regimens in AD.

Studies have also explored the role of IF in other dementia-related conditions, such as vascular dementia, which is a highly prevalent condition associated with cognitive impairments. In a study by Vismitha Rajeev and colleagues, IF was shown to reduce neurovascular damage by reducing metalloproteinase activation and oxidative stress, leading to fewer neuronal deaths in a male C57BL/6NTac mouse model of the disease [21].

IF has also shown efficacy in preclinical models of cognitive impairment associated with neuroinflammation. Indeed, an acute alteration in attention, consciousness, and cognition, with a reduced ability to focus attention, is commonly observed in older adults, typically in response to triggers such as infections or pain [22]. This syndrome is named delirium in the literature and it is associated with an increased risk of developing dementia [22]. Research by Andrea R. Vasconcelos and colleagues has demonstrated that IF improved cognitive function and reduced inflammation in rats subjected to intravenous treatment with lipopolysaccharide [23].

Another common source of cognitive impairment arises from dementia with Lewy bodies and Parkinson’s disease dementia. In a study by Zhi-Lan Zhou and colleagues, IF attenuated the loss of dopaminergic neurons in the substantia nigra in a 1-methyl-4-phenyl-1,2,3,6-tetrathydropyridine-induced Parkinson’s disease mouse model [24]. The effects of IF appear to be mediated by a reduction in neuroinflammation and alterations in the gut microbiome [24].

As for clinical data on the efficacy of IF in cognitive issues, a recent systematic review by Helen Senderovich and colleagues points out that the available data remain inconclusive [25]. Nevertheless, the overall safety of this approach in the studies that were carried out and some promising findings, especially in patients with mild cognitive impairment and mild forms of dementia, were highlighted [26].

At our center at the University of Genoa (Genoa, Italy), together with the Geriatric Clinic of the University of Perugia (Perugia, Italy), a single-blind randomized and placebo-controlled phase I/II clinical trial evaluating the FMD in patients with amnestic mild cognitive impairment or mild forms of AD is currently underway [27]. This study involves patients with good nutritional status and utilizes monthly cycles of FMD, supplemented with compounds known for their neuroprotective, anti-inflammatory, and antioxidant properties. Supplements are also administered throughout the study, including in the refeeding phase following fasting-mimicking diet cycles, to minimize the risk of malnutrition. The primary endpoint of this clinical trial is to assess the feasibility and safety of this dietary regimen in the test population, while secondary objectives include evaluating its efficacy in slowing cognitive decline and on AD and inflammatory biomarkers. Preliminary data published thus far suggest that monthly cycles of the FMD have been feasible and generally safe in patients with mild cognitive impairment and mild AD [14].

In conclusion, although IF regimens have shown promising results in preclinical studies with cognitive disorder models, more research is needed to delve into their mechanisms of action and to verify their efficacy in humans. Much also remains to be understood with respect to possible differences in the mechanisms of action and efficacy of different types of IF (e.g., time-restricted feeding vs. periodic fasting). Comparison studies in different preclinical disease models might prove useful to this end. Finally, as a note of caution, we remind the reader that subjects with neurodegenerative disorders (including their prodromic forms) are at high risk of malnutrition [28]. This remains a major concern in the implementation of these dietary approaches in the clinic. Thus, clinical studies of IF in patients with these types of conditions should select patients carefully and make sure that they are properly followed in terms of their nutritional status. Since the benefits of IF in neurodegenerative diseases remain to be demonstrated, while there remain serious concerns that a patient with dementia could easily become malnourished through IF, this type of diet should only be prescribed to patients with neurodegenerative conditions within clinical trials.
